# 2D Porphyrinic Metal-Organic Frameworks Featuring Rod-Shaped Secondary Building Units

**DOI:** 10.3390/molecules26102955

**Published:** 2021-05-16

**Authors:** Rory Elliott, Aoife A. Ryan, Aviral Aggarwal, Nianyong Zhu, Friedrich W. Steuber, Mathias O. Senge, Wolfgang Schmitt

**Affiliations:** 1 School of Chemistry & AMBER Centre, Trinity College, University of Dublin, Dublin, Ireland; relliot@tcd.ie (R.E.); aviralaggarwal23@gmail.com (A.A.); zhun@tcd.ie (N.Z.); steuberf@tcd.ie (F.W.S.); 2 School of Chemistry, Chair of Organic Chemistry, Trinity Biomedical Science Institute, 152-160 Pearse Street, Trinity College, The University of Dublin, Dublin, Ireland; ryana16@tcd.ie; 3 Focus Group—Molecular and Interfacial Engineering of Organic Nanosystems, Institute for Advanced Study (TUM-IAS), Technical University of Munich, Lichtenberg-Str. 2a, 85748 Garching, Germany; mathias.senge@tum.de

**Keywords:** metal-organic framework, MOF, 2D MOF, 2D materials, rod MOF, Porphyrin MOF, Porphyrinoids, coordination chemistry

## Abstract

Metal-organic frameworks (MOFs) encompass a rapidly expanding class of materials with diverse potential applications including gas storage, molecular separation, sensing and catalysis. So-called ‘rod MOFs’, which comprise infinitely extended 1D secondary building units (SBUs), represent an underexplored subclass of MOF. Further, porphyrins are considered privileged ligands for MOF synthesis due to their tunable redox and photophysical properties. In this study, the Cu^II^ complex of 5,15-bis(4-carboxyphenyl)-10,20-diphenylporphyrin (H_2_**L**-Cu^II^, where H_2_ refers to the ligand’s carboxyl H atoms) is used to prepare two new 2D porphyrinic rod MOFs PROD-1 and PROD-2. Single-crystal X-ray analysis reveals that these frameworks feature 1D Mn^II^- or Co^II^-based rod-like SBUs that are coordinated by labile solvent molecules and photoactive porphyrin moieties. Both materials were characterised using infrared (IR) spectroscopy, powder X-ray diffraction (PXRD) spectroscopy and thermogravimetric analysis (TGA). The structural attributes of PROD-1 and PROD-2 render them promising materials for future photocatalytic investigations.

## 1. Introduction

Over the last decades, MOFs have attracted substantial scientific attention [1,2]. Members of this emerging class of modular, metallo-supramolecular polymeric materials can be conceptualised as repeating combinations of inorganic secondary building units (SBUs or ‘nodes’) that are bridged by multitopic organic ligands (or ‘linkers’) [3]. This arrangement gives infinitely extended multidimensional framework structures with long-range order and high crystallinity [4]. As MOFs demonstrate chemical tunability and high surface areas, they are versatile materials with the potential to advance technologies to tackle several substantial scientific challenges [5,6].

To date, tens of thousands of MOFs have been reported in the literature [7,8,9,10,11]. The vast majority of these structures, including the archetypal frameworks MOF-5 [Zn_4_O(BDC)_3_] (BDC = 1,4-benzene dicarboxylate) and HKUST-1 [Cu_3_(BTC)_2_(H_2_O)_3_] (HKUST = Hong Kong University of Science and Technology, BTC = benzene-1,3,5-tricarboxylate), comprise discrete SBUs [12,13]. Topologically, these frameworks can be understood by abstracting their sub-components as simple geometric shapes such as triangles, squares, tetrahedra, etc., which interconnect to yield a network structure [14,15].

The inimitable modularity and surface areas of MOF materials grants them broad applicability in areas such as gas storage, molecular separation, drug delivery, sensing, and spintronics [16,17,18,19,20,21]. MOFs can accommodate well-defined, physically separated redox-active sites, which also makes them attractive compounds for catalytic applications [22,23,24,25,26]. Frameworks featuring open metal sites (non-coordinatively saturated inorganic nodes) or labile coordinated solvent moieties are particularly promising for catalysis, as these aspects allow substrates to bind with the metal ions of a MOF’s SBU [27,28]. Alternatively, catalytic processes can occur at redox-active metal ions that are embedded within the linkers of certain MOFs, for example porphyrin-based MOFs [29,30]. MOFs are also prime materials for a myriad of electrochemical technologies such as photovoltaics, fuel cells, batteries and supercapacitors [31]. However, porous 3D MOFs constructed from redox-inactive linkers are typically electronically insulating, which limits their suitability for these applications [32,33].

2D MOFs or coordination polymers with layered architectures are a subclass of MOF with distinctive dimensional-dependent characteristics, including exposed surface sites and good flexibility and mechanical stability [34]. The layered architectures of these MOFs can give rise to exceptional electronic, optical and magnetic properties [35,36,37]. Moreover, 2D MOFs often exhibit favourable conductivities, as lower dimensional systems limit charge carrier scattering [38]. Their unique attributes make layered metal-organic materials promising systems for catalysis, conductive devices, and “smart membranes” [39,40,41].

Among the various organic ligands used in MOF synthesis, porphyrins and metalloporphyrins are advantageous due to their unique photophysical and redox properties [30]. These tetrapyrrolic macrocycles are ubiquitous in nature, where they execute essential functions for fundamental biological processes including catalysis, photosynthesis, and gas transport [42,43]. Introducing coordinating carboxylic acid or pyridyl functional groups at a porphyrin’s meso positions allows a macrocycle to be incorporated within a MOF as a rigid, functional linker [44]. In 1991, Robson and co-workers reported the first porphyrinic MOF, which was prepared in a reaction between [5,10,15,20-tetrakis(4-pyridyl)porphyrinato]palladium(II) (MTPP-Pd^II^) and Cd(NO_3_)_2_·4H_2_O in a mixture of boiling MeOH and H_2_O. This 3D framework, formulated as [Cd^II^_2_(MTPP-Pd^II^)(NO_3_)_4_(H_2_O)_4_]·5H_2_O, features mononuclear Cd^II^ nodes that are bridged by palladium tetrapyridyl porphyrin linkers [45]. Subsequent to the discovery of this prototypal porphyrinic framework, significantly more porphyrin-based MOFs have been developed with a wide range of potential applications such as molecular separation, light-harvesting, and photocatalysis [46,47,48,49,50,51,52,53]. The facile tunability of metalloporphyrins has expedited this effort, as simply substituting the central metal ion or β-pyrrole position of a porphyrin yields a linker that can impart improved functionality to a framework.

In contrast to MOFs constructed from finite SBUs, rod MOFs represent a subclass of frameworks with infinitely extended 1D nodes containing chains of periodically repeating metal ions linked by polytopic ligands [54,55,56]. Rod MOFs afford significant advantages over other MOFs, including a lower tendency to form interpenetrated networks and a higher propensity to stabilise open metal sites. Despite this, rod MOFs remain relatively underexplored in the literature [57,58].

In natural light-harvesting systems, highly ordered supramolecular architectures funnel solar energy towards a reaction centre [59,60]. Analogously to biological systems, the regular arrays of proximally positioned π-conjugated porphyrin ligands in porphyrinic MOFs can promote long-range charge transport via networks of π–π stacking interactions [61,62,63,64,65]. Hupp et al. recently investigated exciton migration in two porphyrinic MOFs using fluorescence quenching experiments, revealing that these frameworks facilitate long range anisotropic energy transfer over up to ca. 45 porphyrin struts [46]. This study suggests that porphyrin-based MOFs may be useful bioinspired materials for photoelectrochemical applications, for example as thin film electrode coatings within artificial photosynthetic devices [66,67,68,69]. Similarly, light-harvesting porphyrins and related metal-organic materials with favourable charge transport characteristics can be applied in dye-sensitized or perovskite solar cells to increase the efficiencies of such energy conversion devices [70,71,72,73,74,75].

In this study, two novel porphyrinic rod MOFs [Mn^II^(L-Cu^II^)(MeOH)_2_]·DEA·MeOH (PROD-1, DEA = (*N*,*N*-diethylacetamide) and [Co^II^(L-Cu^II^)(DEA)]·8MeOH (PROD-2) are reported. These compounds comprise distinct, infinitely extended rod-shaped Mn^II^- or Co^II^-based SBUs that are connected by porphyrin ligands, giving rise to 2D sheet architectures. Both PROD-1 and PROD-2 are characterised using single-crystal X-ray diffraction, infrared (IR) spectroscopy, powder X-ray diffraction (PXRD) spectroscopy and thermogravimetric analysis (TGA). Ultimately, due to the presence of specific structural motifs in these frameworks, some potential applications of these materials are discussed.

## 2. Results and Discussion

### 2.1. Synthesis of Metalloporphyrin Rod MOFs

The ditopic porphyrin 5,15-bis(4-carboxyphenyl)-10,20-diphenylporphyrin (H_4_L) was selected to synthesise MOFs endowed with the unique electronic and photophysical properties of porphyrins. This choice was rationalised due to the rich chemical diversity of MOFs constructed from other, more rudimentary dicarboxylate linear linkers, and because at the time of writing only two MOFs reported in the Cambridge Structural Database (CSD, Version 5.41) contain this underexplored porphyrin [76,77,78,79,80,81,82]. Additionally, the central cavity of H_4_L can accommodate an array of metal ions, which facilitates further tuning of frameworks constructed using this linker [83,84]. Considering the promising light-harvesting [85] and catalytic [86] properties of comparable Cu^II^ metalloporphyrin complexes, and the low cost and toxicity of Copper, [5,15-bis(4-carboxyphenyl)-10,20-diphenylporphyrinato]copper(II) (H_2_L-Cu^II^) was prepared in moderate yield (see experimental for details) [87,88].

**Figure 1:**
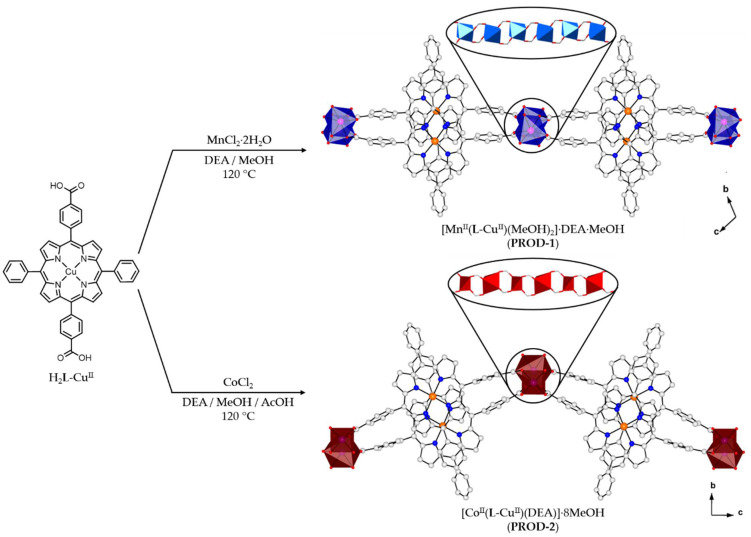
H_2_**L**-Cu^II^ was used to prepare two 2D porphyrinic rod MOFs, PROD-1 and PROD-2. Insets show the 1D chain SBUs of PROD-1 and PROD-2. H atoms and solvent molecules are omitted for clarity. Colour scheme: C white, N blue, O red, Cu orange, Mn pink, Co violet. Mn^II^ and Co^II^ coordination environments are represented by blue and red polyhedra, respectively.

H_2_L-Cu^II^ was used to prepare two 2D MOFs [Mn^II^(L-Cu^II^)(MeOH)_2_]·DEA·MeOH (PROD-1) and [Co^II^(L-Cu^II^)(DEA)]·8MeOH (PROD-2) with 1D Mn^II^- or Co^II^-based SBUs, respectively (Figure 1). PROD-1 was prepared by heating H_2_L-Cu^II^ and MnCl_2_·2H_2_O in a mixture of DEA and MeOH to 120 °C for four days in a Teflon-lined stainless-steel autoclave. Slowly cooling this reaction mixture to room temperature afforded the formation of uniform, rod-shaped, crimson crystals of PROD-1. Similarly, PROD-2 was synthesised by heating H_2_L-Cu^II^ and CoCl_2_ in a mixture of DEA, MeOH and acetic acid (AcOH) under solvothermal conditions. After four days, this reaction mixture was slowly cooled to ambient temperature, resulting in the formation of red, plate-shaped crystals of PROD-2. Both products PROD-1 and PROD-2 form reproducibly, in good yields and were of suitable quality for analysis using single-crystal X-ray diffraction.

### 2.2. Crystal Structure of [Mn^II^(L-Cu^II^)(MeOH)_2_]·DEA·MeOH (PROD-1)

**Figure 2:**
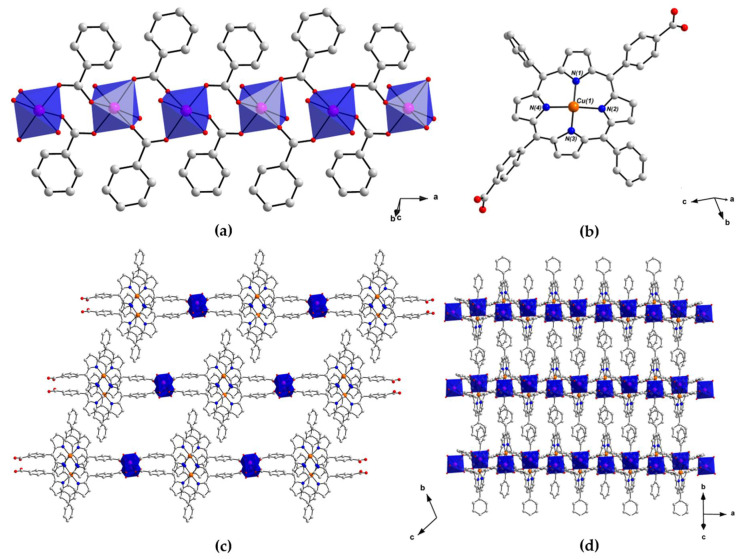
View of the structure of PROD-1 in the crystal, showing (**a**) the MOF’s rod-shaped SBU which comprises an infinite 1D chain of octahedrally coordinated Mn^II^ ions bridged by the *syn–syn* coordinating carboxylate functionalities of (**L**-Cu^II^)^2−^ linkers, (**b**) the linear metalloporphyrin ligand (**L**-Cu^II^)^2−^ and (**c**,**d**) the packing arrangement of PROD-1, highlighting the MOF’s layered architecture. H atoms and solvent molecules are omitted for clarity. Colour scheme: C white, N blue, O red, Mn pink, Cu orange. Mn^II^ coordination environments are shown as blue polyhedra.

The single-crystal X-ray structure of PROD-1 was solved in the triclinic space group $\;P\bar{1}$. This analysis was hampered due to twinned crystals and weak diffraction. However, the data allowed us to establish the structure’s connectivity. PROD-1 comprises stacking 2D sheets, containing 1D rod-like Mn^II^-based SBUs that extend infinitely in the direction of the crystallographic *a*-axis (Figure 2a). The SBUs of PROD-1 are each linked to two other identical inorganic nodes by ditopic (L-Cu^II^)^2−^ ligands (Figure 2b). This connectivity gives rise to 2D sheets which extend with the crystallographic *ac*-plane, and in which rod-shaped SBUs stack in parallel with one another (Figure 2c,d). Neighbouring interconnected inorganic nodes within the 2D sheets are separated by ca. 22 Å.

The asymmetric unit of PROD-1 contains one Mn^II^ centre, one (L-Cu^II^)^2−^ ligand, two coordinated MeOH solvent moieties, and constitutional DEA and MeOH solvent molecules which locate between the small channels that extend between the MOF’s 2D layers. The SBU of PROD-1 comprises an infinite chain of octahedrally coordinated Mn^II^ centres, each of which connect to two adjacent metal ions through four bridging carboxylate moieties that derive from four (L-Cu^II^)^2−^ linkers. The interatomic distance between two Mn^II^ centres within the 1D SBU is ca. 4.6 Å.

**Figure 3:**
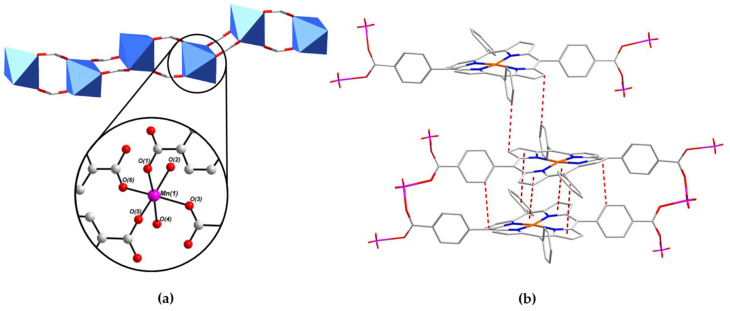
(**a**) The coordination environment of the Mn^II^ ion Mn(1) within the 1D chain SBU of PROD-1, which includes two MeOH solvent-derived O-donors O(2) and O(4), and (**b**) a wireframe representation highlighting π–π stacking interactions which stabilize PROD-1. H atoms have been omitted for clarity. Colour scheme: C white, N blue, O red, Cu orange, Mn pink. Blue polyhedra represent Mn^II^ coordination environments. π–π interactions are highlighted using dashed red lines.

The binding environment of the Mn^II^ centre Mn(1) within the 1D SBU of PROD-1 is shown in Figure 3a. The coordination sphere of Mn(1) comprises four O-donors O(1), O(3), O(5) and O(6) from four distinct *syn*–*syn* bridging, *μ*_2_-*η*^1^:*η*^1^ binding carboxylate functionalities and two O-donors O(2) and O(4) which derive from two ‘*cis*’-coordinated MeOH solvent moieties. The bond distances between Mn(1) and each of the four carboxylate O-donors are within the range 2.15–2.17 Å, whereas the distances from the Mn^II^ ion and the more labile, monodentate MeOH moieties range between 2.21 Å and 2.23 Å. The bond angles surrounding Mn(1) render its coordination geometry a slightly distorted octahedron, and are consistent with values reported for comparable Mn^II^-carboxylate complexes in the literature [89,90].

An extensive network of π–π stacking interactions stabilise this structure, some of which are shown in Figure 3b. For example, T-shaped intersheet π–π stacking interactions between the meso-phenyl and tetrapyrrole moieties of (L-Cu^II^)^2−^ linkers, and parallel-displaced intrasheet π–π stacking interactions between adjacent metalloporphyrins are highlighted. These supramolecular interactions propagate in parallel with the crystallographic *b*-axis and extend the 2D network into a 3D framework. The distances between π–π interacting moieties in PROD-1 are within the range of 3.4–3.8 Å, which is consistent with π–π stacking interactions reported in the literature [91,92].

The porphyrin linkers within PROD-1 adopt saddle-shaped configurations and have staggered meso-functionalities [93]. The bond angles between ‘*trans’*-coordinated pyrrolic N-donors deviate from the ideal square planer angle by up to ca. 8°, whilst the dihedral angles between meso-carboxyaryl and meso-phenyl functional groups are ca. 27(1)° and 43(1)°, respectively. This configuration facilitates stabilisation of the structure through π–π stacking interactions.

(L-Cu^II^)^2−^ is deprotonated at both of its carboxylic acid binding sites, giving the linker a charge of −2. As one ligand is present per Mn^II^ centre in the crystal structure, the overall charge of PROD-1 is balanced. Bond valence sum analysis (BVSA) calculations confirmed the oxidation states of all metal ions in PROD-1.

In the crystal structure of PROD-1, 2D sheets pack densely and porphyrin moieties interdigitate between neighbouring layers. Small, interlayer channels filled with constitutional solvent molecules and infinite 1D *zig-zag* Mn^II^ chains extend in parallel with the crystallographic *a*-axis. The average distance between two adjacent 2D sheets in PROD-1 is ca. 15 Å. The solvent-accessible void volume of PROD-1 was calculated as 250 Å^3^ (accounting for 10.4% of the unit cell volume) using the CCDC-mercury program with a probe radius of 1.2 Å and a grid spacing of 0.7 Å [94].

### 2.3. Crystal Structure of [Co^II^(L-Cu^II^)(DEA)]·8MeOH (PROD-2)

**Figure 4:**
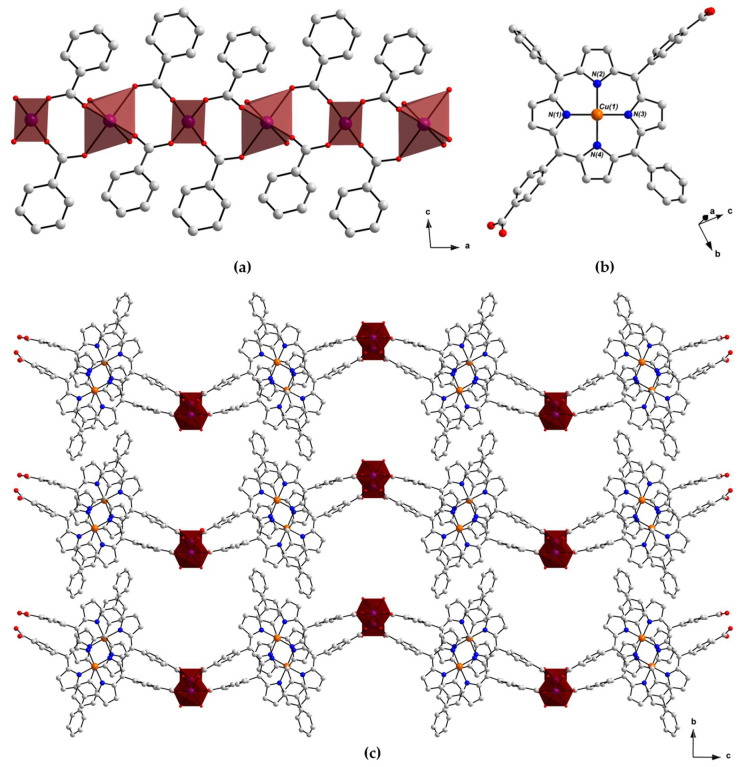
View of the structure of PROD-2 in the crystal, highlighting (**a**) the framework’s 1D rod-shaped SBU comprising an infinite chain of alternating tetrahedrally and octahedrally coordinated Co^II^ ions, (**b**) the linker (L-Cu^II^)^2−^ and (**c**) the packing arrangement of PROD-2. Hydrogen atoms and solvent molecules are omitted for the purpose of clarity. Colour scheme: C white, O red, N blue, Cu orange. Red polyhedra represent Co^II^ coordination environments.

The single-crystal X-ray structure of PROD-2 was solved in the monoclinic space group *P*2/*c*, revealing that this MOF and PROD-1 are structurally alike, as both of these frameworks feature 2-connected rod-shaped SBUs (Figure 4a) and ditopic (L-Cu^II^)^2−^ ligands (Figure 4b). Unlike PROD-1, however, the infinite 1D rod-like node of PROD-2 comprises periodically repeating, alternating tetrahedral and octahedral Co^II^ centres. Interconnected, infinitely extended Co^II^ chains and porphyrin linkers form 2D layers that extend in parallel with the crystallographic *ac*-plane and undulate in the crystallographic *b*-directions due to the alternating coordination geometries of this MOF’s metal ions. The 2D layers of PROD-2 of stack on top of one another and interdigitate, giving rise to the corrugated sheet structure shown in Figure 4c.

The asymmetric unit of PROD-2 contains two distinct Co^II^ centres Co(1) and Co(2) which locate at two-fold rotational axes and have a crystallographic occupancy of ½, one doubly deprotonated (L-Cu^II^)^2−^ ligand and one coordinated DEA solvent molecule. Disordered constitutional solvent molecules which could not be refined in the crystal structure of PROD-2 were masked using the Platon-Squeeze routine [95]. The interatomic distance between Co(1) and Co(2) is 4.5020(14) Å, and the distance between two connected rod-shaped nodes is ca. 22 Å.

**Figure 5:**
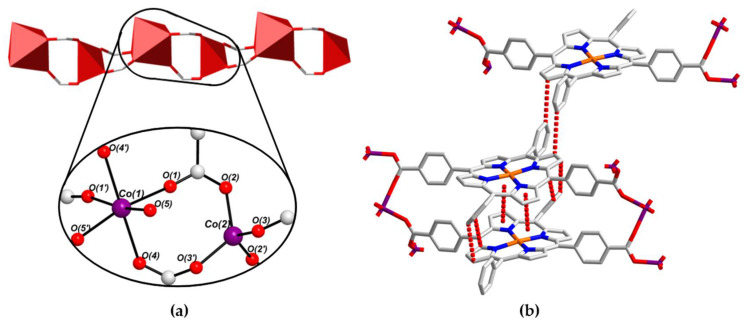
(**a**) Coordination environments of the Co^II^ ions within PROD-2′s 1D SBU, featuring two DEA-derived O-donors O(5) and O(5′), and (**b**) selected π–π stacking interactions that stabilize PROD-2. H atoms are omitted for clarity. Colour scheme: C white, N blue, O red, Cu orange, Co violet. Red polyhedra represent Co^II^ coordination spheres and dashed red lines show π–π stacking.

The binding environments of Co(1) and Co(2) within the *zig-zag* chain SBU of PROD-2 are depicted in Figure 5a. These ions adopt distorted octahedral and distorted tetrahedral coordination geometries, and are each coordinated by four O-donors from four bridging, *μ*_2_*-η*^1^*:η*^1^ binding carboxylate moieties that link adjacent Co^II^ centres into infinite 1D chains. In addition two O-donors, O(5) and O(5′), derived from two labile, ‘*cis*’-coordinated DEA solvent moieties are contained within the coordination sphere of Co(1). The bond distances surrounding the octahedral Co^II^ centre Co(1) are within the range of 2.2626(5)–2.3850(5) Å, whilst the bond distances around the tetrahedrally coordinated Co^II^ ion Co(2) range between 1.9484(4) Å and 1.9604(5) Å. The bond distances and angles surrounding Co(1) and Co(2) are consistent with the values reported for other tetrahedral and octahedral Co^II^ carboxylate complexes in the literature [96,97,98].

As in PROD-1, the porphyrin ligand of PROD-2 adopts a saddle-shaped configuration which facilitates intrasheet π–π stacking interactions between neighbouring (L-Cu^II^)^2−^ moieties (Figure 5b). The extent to which the linkers of PROD-2 are distorted is marginally less than those of PROD-1. Dihedral angles between meso-substituted phenyl, and carboxyaryl functionalities in PROD-2 vary by up to 33.0°, which promotes intersheet T-shaped π–π interactions between nearby porphyrins. The distances between π–π stacking moieties in PROD-2 fall within the range of 3.5–3.8 Å [92].

The porphyrin linker in PROD-2 is doubly deprotonated, and thus has a charge of −2. This charge is balanced by two crystallographically ½ occupied Co^II^ ions Co(1) and Co(2), giving PROD-2 a net charge of 0. BVSA calculations confirmed the assignment of the +2 oxidation state of Co(1), Co(2) and Cu(1). Undulant interdigitated 2D sheets pack densely in the crystal structure, giving PROD-2 its characteristic corrugated conformation. Small, interlayer solvent-accessible channels propagate in parallel with the crystallographic *a*-axis, constituting a solvent-accessible void volume of 452 Å^3^ which corresponds to 8.9% of the MOF’s unit cell (calculated using CCDC-mercury with a probe radius and grid spacing of 1.2 and 0.7 Å, respectively) [94].

PROD-1 and PROD-2 crystallize under comparable reaction conditions and share several structural similarities. Both structures inhabit an intersection in chemical space between 2D, rod and porphyrin-based MOFs, which confers a constellation of characteristics making them desirable for various future technologies. For instance, PROD-1 and PROD-2 contain well-ordered arrays of metalloporphyrin linkers and are stabilised by π–π stacking interactions [99]. This could give rise to light-harvesting or energy transfer properties that are advantageous for photo- or electrochemical applications including photovoltaics or photocatalysis [100]. PROD-1 and PROD-2 are particularly promising for catalytic applications, as their layered architectures, 1D channels and rod-shaped SBUs support high concentrations of exposed labile coordination solvent sites. Moreover, the presence of photoactive porphyrin linkers that coordinate directly to potentially redox-active Mn^II^ and Co^II^ centres in PROD-1 and PROD-2 could give rise to photocatalytic activity. Finally, the tunable and fully noble metal-free nature of these compounds is advantageous, as it facilitates facile modifications and lowers the investment costs associated with these MOFs.

### 2.4. Physicochemical Characterisation of PROD-1 & PROD-2

The IR spectra of PROD-1 (Figure S1) and PROD-2 (Figure S2) exhibit characteristic signals confirming the presence of the porphyrin ligand (L-Cu^II^)^2−^. For example, several signals centred around 3000 cm^−1^ are attributed to aromatic C–H stretching vibrations of the metalloporphyrin linkers in both MOFs. In addition, bands at ca. 1540 and 1380 cm^−1^ are assigned to asymmetric and symmetric stretching vibrational modes, respectively, of the *μ*^2^*-η*^1^*:η*^1^ bridging carboxylate moieties of PROD-1 and PROD-2 [101]. Weak signals at around 1280 cm^−1^ can be attributed to C–N stretching vibrations of the MOF’s (L-Cu^II^)^2−^ linkers. Furthermore, sharp signals at 1001 and 994 cm^−1^ may arise due to in-plane vibrations (ring breathing) of the porphyrin macrocycles of PROD-1 and PROD-2, respectively [102]. Finally, bands at ca. 1600 cm^−1^ can be attributed to C=O stretching vibrations from the coordinated and constitutional DEA solvent molecules within PROD-1 and PROD-2 [103].

The thermal stabilities of PROD-1 and PROD-2 were investigated using TGA by heating these compounds from 30–800 °C under N_2_. The TGA trace of PROD-1 (Figure S3) reveals that when heated from 25–80 °C, the sample undergoes a weight loss of 2.5% which can be attributed to the loss of one constitutional MeOH solvent molecule (calculated: 3.1%). It is likely that some solvent of crystallisation was lost from the framework prior to this analysis. An additional mass loss of 5.2% is observed as the sample is heated from 80–300 °C, which can be accounted for by the loss of a DEA coordination solvent molecule from PROD-1 (calculated: 6.2%). Further heating of the sample from 300–455 °C was associated with a further mass loss of 11.0%, which results from the loss of one constitutional DEA solvent molecule from PROD-1 (calculated: 11.2%). Two additional thermogravimetric steps are observed between 455–540 °C and above 540 °C, which can be attributed to the decomposition of the MOF’s organic components and to the formation of metal oxide species, respectively.

The TGA trace of PROD-2 (Figure S4) shows that the structure degrades in several distinct thermogravimetric steps. As the sample is heated from 30–105 °C a mass loss of 20.6% is observed. This decrease in mass can be attributed to the loss eight constitutional MeOH solvent molecules from PROD-2 (calculated: 21.5%). When PROD-2 is heated further from 105–360 °C, a weight loss of 9.3% is observed. This weight loss is associated with the loss of one coordinated DEA molecule from PROD-2 (calculated: 9.7%). Heating the sample from 360–500 °C is associated with the decomposition of the framework’s organic ligands. Finally, the formation of metal oxide species occurs as the sample is heated above 500 °C. These TGA experiments reveal that both PROD-1 and PROD-2 are thermally stable up to ca. 350 °C, as the MOF’s porphyrin linkers start to decompose above this temperature. As the disordered constitutional solvent molecules of PROD-2 could not be modelled crystallographically, interpretation of these data enabled the assignment of this compound’s molecular formula [Co^II^(L-Cu^II^)(DEA)]·8MeOH. Upon desolvation, both PROD-1 and PROD-2, do not take up significant quantities of N_2_.

PXRD patterns of PROD-1 (Figure S5) and PROD-2 (Figure S6) were measured and compared with simulated PXRD patterns that were calculated from the corresponding compound’s single-crystal X-ray diffraction data to evaluate the phase purity of the prepared samples. The experimentally obtained PXRD pattern of PROD-1 agrees well with its simulated pattern. Discrepancies for PROD-2 may result from structural changes due to rapid sample desolvation prior to or during PXRD analysis and crystal orientation effects. 

## 3. Materials and Methods 

### 3.1. Reagents & Analytical Methods

All chemicals and solvents were of reagent grade and used as received without further purification unless otherwise stated. Single-crystal X-ray structural analyses were performed on a Bruker SMART APEX CCD diffractometer with a Cu-Kα X-ray source (λ = 1.54184 Å). The omega scan method was used to collect either a full sphere or hemisphere of data for each crystal with a detector to crystal distance of either 5 or 6 cm at temperatures of 100 or 215 K. Data were collected, processed, and corrected for Lorentz and polarisation effects using SMART [104] and SAINT-PLUS [105] programs. The structures were solved using direct methods with the SHELXTL [106] software package. All non-hydrogen atoms were refined anisotropically. Hydrogen atom positions were assigned using a riding model with appropriately fixed isotropic thermal parameters. Solvent-accessible void volumes were calculated using the ‘voids’ tool in Mercury (CCDC) using a probe radius of 1.2 Å and a grid spacing of 0.7 Å [94]. Crystallographic information files for PROD-1 and PROD-2 can be obtained free of charge from the Cambridge Crystallographic Data Centre via www.ccdc.cam.ac.uk/data_request/cif using the accession identifiers CCDC-2073036 and CCDC-2065629, respectively. For the structural refinement of PROD-1, the DFIX constraint was applied to the bonds of an identified coordinated *N*,*N*-diethylacetamide (DEA) molecule due its disorder in the structure. Common C–C, C–N and C–O bond lengths, characteristic for DEA molecules were applied. Additionally, a FLAT command, restraints some of the atoms of the DEA molecule to lie in a common plane. The approach resulted in convergence upon least-square refinements. Further DEA and MeOH solvent molecules were located in the voids of the structure and their occupancies were refined to achieve convergence. The occupancies <1 are caused by solvent loss during mounting and data collection or disorder of the solvent molecules whereby parts of the disordered positions could not be located. The twin character of the crystals of PROD-2 was noted during the crystallographic data collection. A careful selection of reflection spots was required prior to indexing and integration. The HKLF5 command was applied to the refinement. This approach allowed us to solve the structure. Finally, the Platon twin routine was applied to further resolve the degree of twinning, leading to the reported quality values. The Platon-Squeeze routine was applied due to the diffuse electron density that results from highly disordered solvent molecules located in the voids of the structure. The solvent-accessible void volume accounts to 1143 Å^3^ and 272 electrons. This electron contribution stems from solvent molecules, i.e., methanol which was used in the synthesis. The TGA analysis is consistent with the crystallographic data. Based on this analysis, 8 constitutional MeOH molecules were assigned to the structure.

IR spectra were recorded using a PerkinElmer Spectrum One FT-IR spectrometer equipped with a universal ATR sampling accessory. Data were collected and processed using Spectrum v5.0.1 (2002 PerkinElmer Instrument LLC) software. The scan rate was 16 scans per second with a resolution of 4 cm^−1^ in the range 4000–650 cm^−1^. Standard abbreviations were used to describe signal intensities: s, strong; m, medium; w, weak; br, broad. Thermogravimetric analysis (TGA) data were collected using a simultaneous SDT thermal analyser at a heating rate of 5 °C min^−1^ under a N_2_ atmosphere (N_2_ flow rate = 0.06 L min^−1^). PXRD data were collected at room temperature with a Bruker D2 Phaser diffractometer equipped with a CuKα X-ray source. Simulated PXRD patterns were calculated from the single-crystal X-ray data of PROD-1 and PROD-2 using the CCDC-Mercury program (Cambridge, UK) [94]. ^1^H NMR spectra were recorded on a Bruker DPX 400 spectrometer operating at 400.13 MHz. Samples were analysed in deuterated solvents that are listed for each spectrum. Standard abbreviations are used for spectral assignments: s, singlet; d, doublet; t, triplet; m, multiplet; br, broad; J, coupling constant. UV-Vis spectra were recorded in the range 300–800 nm on a Cary Scan spectrophotometer at 20 °C using disposable cells with a path length of 1 cm.

### 3.2. Synthesis of the Porphyrin Ligand H_2_L-Cu^II^

The dicarboxylic acid porphyrin H_2_L-Cu^II^ was prepared in four steps, beginning with the synthesis of 5-(4-carbomethoxyphenyl)dipyrromethane, according to adapted procedures described by Lindsey et al. [107] and Meindl et al. [108] Under an inert atmosphere and while shielding from light, trifluoroacetic acid (TFA, 0.23 mL 0.343 mmol) was added to a solution of methyl-4-formyl benzoate (5.00 g, 30.4 mmol) in freshly distilled pyrrole (150 mL, 2.162 mol), before stirring the solution for 3 h. NaOH (3.60 g, 0.090 mol) was then added, and the reaction was stirred for a further hour. Following this, the reaction mixture was filtered before concentrating the filtrate by evaporating the solvent under reduced pressure, while excess pyrrole was recovered for later use. The crude product obtained was subsequently purified by silica gel column chromatography using a mixture of hexane, dichloromethane (DCM), ethyl acetate and triethylamine (TEA) in a ratio of 4:2:1:0.05 (vol/vol) as the eluting solvent before washing with cold ethyl acetate yielding 5-(4-carbomethoxyphenyl)dipyrromethane as a white powder. Yield: 4.26 g (50%). ^1^H NMR (400 MHz, CD_3_CN): δ (ppm) = 8.94 (m, 2H, NH), 7.92 (m, 2H, aryl-H), 7.32 (m, 2H, aryl-H), 6.66 (m, 2H, pyrrole-H), 6.02 (m, 2H, pyrrole-H), 5.77 (m, 2H, pyrrole-H), 5.52 (s, 1H, CH), 3.85 (s, 3H, CH_3_).

Next, under an inert atmosphere and darkness, benzaldehyde (3.6 mL, 35.3 mmol) was added to a solution of 5-(4-carbomethoxyphenyl)dipyrromethane (9.60 g, 34.5 mmol) in dry DCM (3.3 L). To this solution, TFA (6.6 mL) was added dropwise over 1 min. The reaction mixture was then stirred for 3 h before adding *p*-chloranil (12.48 g, 50.7 mmol) and stirring overnight. Following this, TEA (6.6 mL) was added to quench the reaction before removing the solvent under reduced pressure. The crude product was then dry loaded onto silica and purified using silica gel column chromatography with CH_3_Cl as the eluting solvent. A purple powder consisting of several different methoxy ester-substituted porphyrins was obtained as the second purple band after 5,10,15,20-tetraphenylporphyrin. This powder was then dry loaded on to silica and purified using silica gel column chromatography with DCM and hexane in a ratio of 2:1 (vol/vol) as the eluent, giving the desired diester porphyrin in the third purple band. Evaporation of the solvent under reduced pressure yielded 5,15-bis(4-carbomethoxyphenyl)-10,20-diphenylporphyrin as a purple powder. Yield: 0.6 g (17%). ^1^H NMR (400, MHz CDCl_3_): δ (ppm) = 8.80 (m, 4H, pyrrole-H), 8.75 (m, 4H, pyrrole-H), 8.45 (d, 4H, benzyl-H, *J* = 9.2 Hz), 8.28 (d, 4H, benzyl-H, *J* = 6.9 Hz), 8.20 (d, 4H, aryl-H, *J* = 9.2 Hz), 7.78 (m, 6H, aryl-H), 4.12 (s, 6H, CH_3_), −2.78 (br, s, 2H, pyrrole-H). UV-Vis (DMF): λ_max_/nm (ε/L mol^−1^ cm^−1^) = 415 (1.8 × 10^5^, Soret band, π–π*), 513 (9.0 × 10^3^, Q band, π–π*), 546 (4.0 × 10^3^, Q band, π–π*), 590 (3.4 × 10^3^, Q band, π–π*), 645 (2.9 × 10^3^, Q band, π–π*). MS (MALDI-TOF): Found *m/z* = 730.2417. Calculated *m/z* = 730.2580 for [C_48_H_34_N_4_O_4_].

Following this, 5,15-bis(4-methoxycarbonylphenyl)-10,20-diphenylporphyrin (600 mg, 0.821 mmol) was dissolved in CHCl_3_ (100 mL) and heated to 70 °C. Cu(OAc)_2_ monohydrate (1.624 g, 8.210 mmol) dissolved in MeOH (10 mL) was then added to the heated solution and the reaction was stirred at 70 °C for 3 h. Upon complete consumption of the starting material, monitored via TLC analysis (CH_2_Cl_2_: hexanes, 2:1, *v*/*v*), the reaction mixture was washed with saturated NaHCO_3_, water and brine (2 × 100 mL each), extracting with CH_2_Cl_2_. The organic extracts were dried over Na_2_SO_4_, filtered and the solvents were removed in vacuo. The red residue {5,15-bis(4-methoxycarbonylphenyl)-10,20-diphenylporphyrinato}copper(II)} was used without further purification in the ester hydrolysis step. (5,15-Bis(4-methoxycarbonylphenyl)-10,20-diphenylporphyrinato)copper(II) (approximately 600 mg) was dissolved in THF (50 mL) and to this solution KOH (12 g in 50 mL H_2_O) was added. The reaction mixture was heated to 80 °C and was stirred at this temperature for 18 h. Upon complete consumption of the starting material, the reaction mixture was cooled to ambient temperature before acidification to pH 6 using 1M HCl. The product was extracted using CH_2_Cl_2_ and the organic extracts were combined, and the solvents were removed in vacuo. The red residue was triturated with CHCl_3_ and filtered to give a red solid which was subsequently transferred to a 250 mL RBF, suspended in CHCl_3_ and the solvent was removed *in vacuo* to yield a red residue (600 mg, 0.785 mmol, 96%). Mp >300 °C; R_f_ (CH_2_Cl_2_: hexane: MeOH 1:1:0.02 *v*/*v*/*v*): 0.63; UV-vis (MeOH): λ_max_ (log *ε*) = 412 (5.35), 538 (4.02) nm; HRMS (MALDI): *m/z* = 764.2849 calculated for C_46_H_28_N_4_O_4_Cu: found; 763.1432.

### 3.3. Synthesis of [Mn^II^(L-Cu^II^)(MeOH)_2_]·DEA·MeOH (PROD-1)

H_2_L-Cu^II^ (15.0 mg, 19.7 μmol) and MnCl_2_·2H_2_O (3.20 mg, 19.8 μmol) were dissolved in *N*,*N*-diethylacetamide (DEA) (11.0 mL) and MeOH (6.0 mL) by sonicating the mixture for 30 min. The resulting solution was then transferred into a Teflon-lined stainless-steel autoclave and heated to 120 °C for 4 days, before slowly cooling to ambient temperature. This afforded the formation of crimson, rod-shaped crystals of PROD-1 which were of suitable quality for analysis using single-crystal X-ray diffraction. Yield: 4.78 mg (24%). FT-IR: υ_max_ = 2978 (br, w), 1607 (m), 1581 (s), 1536 (m), 1487 (w), 1366 (vs), 1282 (w), 1210 (w), 1175 (w), 1012 (w), 1001 (s), 831 (w), 796 (s), 697 (s) cm^−1^.

### 3.4. Synthesis of [Co^II^(L-Cu^II^)(DEA)]·8MeOH (PROD-2)

H_2_L-Cu^II^ (1.2 mg, 1.56 μmol) and CoCl_2_ (1.5 mg, 11.5 μmol) were dissolved in *N*,*N*-diethylacetamide (DEA) (1.0 mL) and MeOH (0.5 mL) by sonicating for 30 min. Following this, a drop of acetic acid was added to the reaction mixture and the solution was transferred into a small glass vial, which was subsequently placed inside a sealed Teflon-lined stainless-steel autoclave and heated to 120 °C for 4 days. Slow cooling of the reaction mixture to room temperature over 24 h afforded the formation of red, plate-shaped crystals of PROD-2 which were suitable for single-crystal X-ray diffraction studies. Yield: 0.6 mg (39%). FT-IR: υ_max_ = 2969 (vbr, m), 1594 (s), 1548 (m), 1373 (s), 1334 (s), 1276 (m), 1204 (w), 1181 (w), 1067 (w), 994 (s), 832 (w), 797 (s), 773 (s), 699 (s) cm^−1^.

## 4. Conclusions

In conclusion, we report two new 2D porphyrinic MOFs with rod-shaped SBUs, PROD-1 and PROD-2, which are rare examples of frameworks featuring the underexplored linker H_2_**L**-Cu^II^. Single-crystal X-ray diffraction studies show that the contrasting coordination geometries found within the MOFs’ 1D metal chain SBUs give rise to either planar or corrugated sheet configurations. PROD-1 and PROD-2 are thermally stable up to ca. 350 °C and exhibit extensive networks of π–π stacking interactions. The present structures are intriguing supramolecular substances which span several subclasses including 2D, porphyrinic and rod MOFs. Their unique attributes render them hopeful compounds for a wide range of future applications, and their structural relationship to Nature’s paragon H_2_O splitting Mn complex in PS II, suggests that PROD-1 and PROD-2 might ultimately find use within bioinspired artificial photosynthetic systems [109].

## Data Availability

Crystallographic information files for PROD-1 and PROD-2 can be obtained free of charge from the Cambridge Crystallographic Data Centre via www.ccdc.cam.ac.uk/data_request/cif using the accession identifiers CCDC-2073036 and CCDC-2065629, respectively.

## Supplementary Materials

Electronic supporting information containing crystallographic tables and physicochemical characterisation data for PROD-1 and PROD-2, and crystallographic information files (CIFs) are available online.

## Sample Availability

Samples of the compounds are not available from the authors.
