# Supplementary material for: 2D Porphyrinic Metal-Organic Frameworks Featuring Rod-Shaped Secondary Building Units
Source: Molecules. 2021 May 16;26(10):2955. doi: 10.3390/molecules26102955 (PMC8156857; doi:10.3390/molecules26102955)
Supplement: Supplementary file 1 [file molecules-26-02955-s001.zip › molecules-1187738-supplementary.pdf]

Supporting Information

2D Porphyrinic Metal-Organic Frameworks Featuring Rod-Shaped Secondary Building Units

Rory Elliott[1], Aoife A. Ryan[2], Aviral Aggarwal[1], Nianyong Zhu[1], Friedrich W. Steuber[1], Mathias O. Senge[3] and Wolfgang Schmitt[1,*]

[1] School of Chemistry & AMBER Centre, Trinity College Dublin, University of Dublin, Ireland; relliot@tcd.ie (R.E.), aviralaggarwal23@gmail.com (A.A.), steuberf@tcd.ie (F.S.) zhun@tcd.ie (N.Z.),

[2] School of Chemistry, Chair of Organic Chemistry, Trinity Biomedical Science Institute, 152-160 Pearse Street, Trinity College, University of Dublin, Ireland; ryana16@tcd.ie (A.R.)

[3] Institute for Advanced Study (TUM-IAS), Technical University of Munich, Focus Group – Molecular and Interfacial Engineering of Organic Nanosystems, Lichtenberg-Str. 2a, 85748 Garching, Germany; mathias.senge@tcd.ie (M.S.)

*Correspondence to: schmittw@tcd.ie

Table of Contents

Supporting Figures

Supporting Figure 1..........S2

Supporting Figure 2..........S2

Supporting Figure 3..........S3

Supporting Figure 4..........S3

Supporting Figure 5..........S4

Supporting Figure 6..........S4

Supporting Figure 7..........S5

Supporting Figure 8..........S5

Supporting Figure 9..........S6

Supporting Figure 10..........S6

Supporting Tables

Supporting Table 1..........S7

Supporting Table 2..........S8

Supporting Table 3..........S10

Supporting Table 4..........S10

1. Infrared Spectroscopy

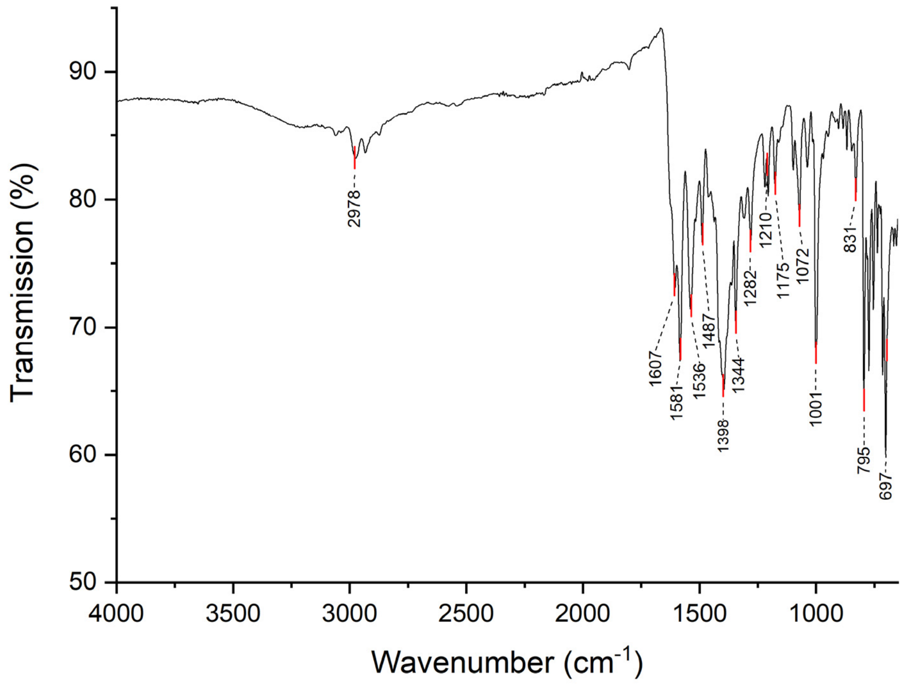

Figure S1. Infrared spectrum of **PROD-1**.

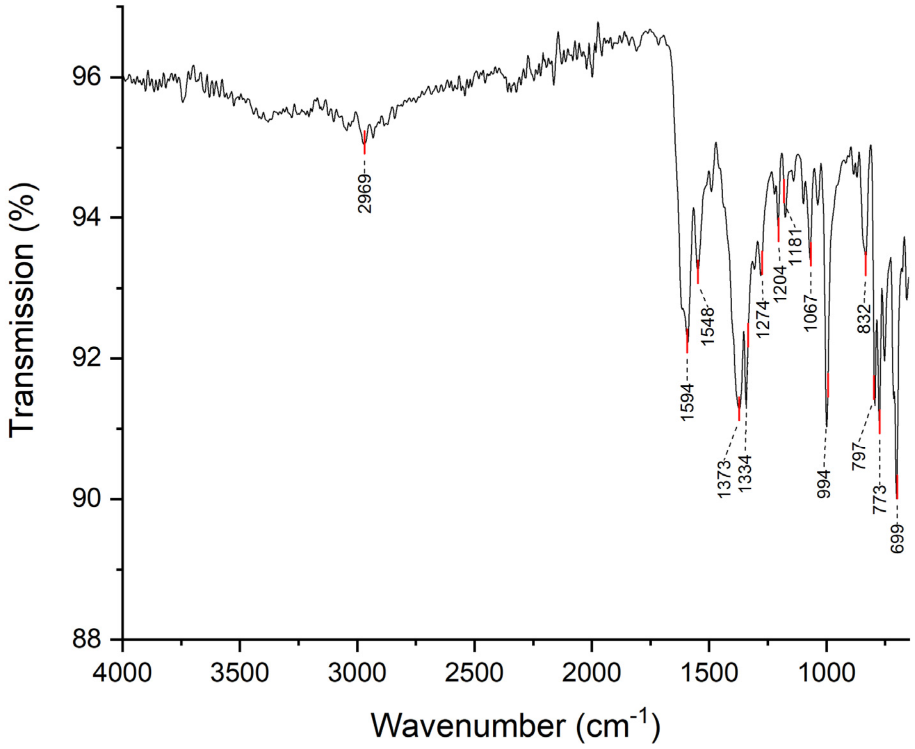

Figure S2. Infrared spectrum of **PROD-2**.

2. Thermogravimetric Analysis

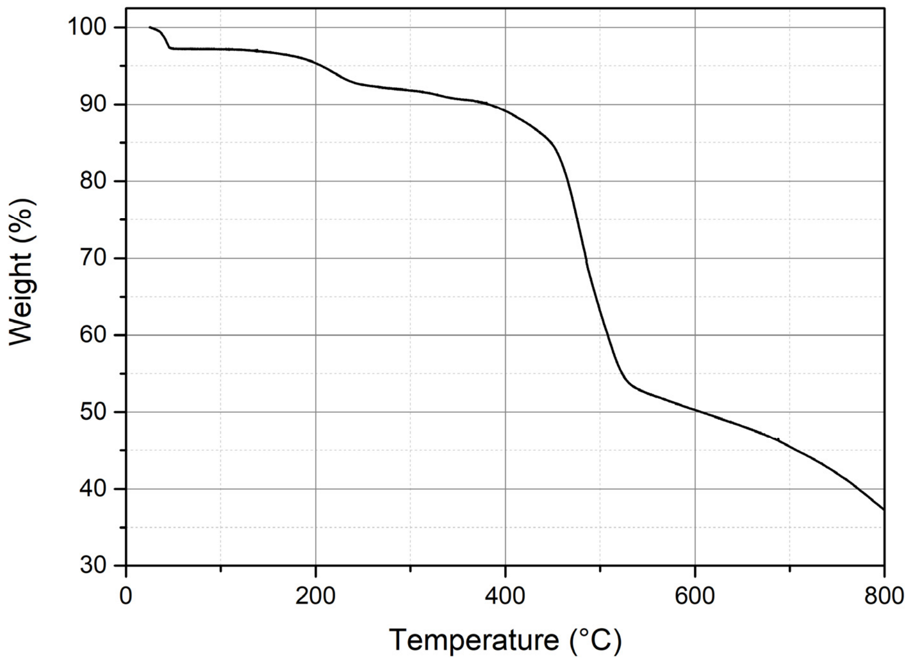

Figure S3. TGA trace of **PROD-1**.

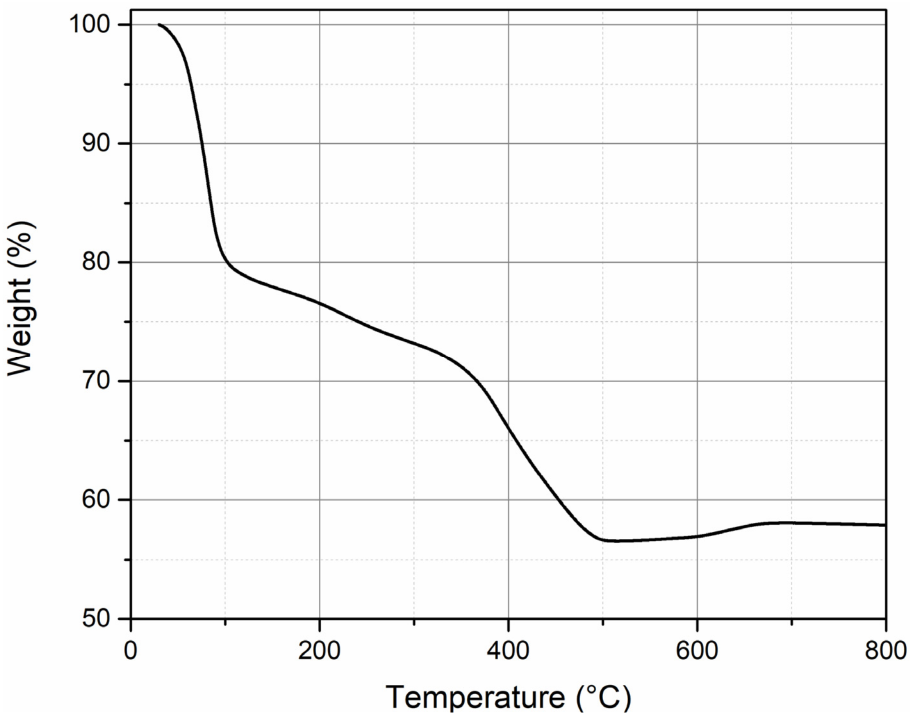

Figure S4. TGA trace of **PROD-2**.

3. Powder X-ray Diffraction (PXRD)

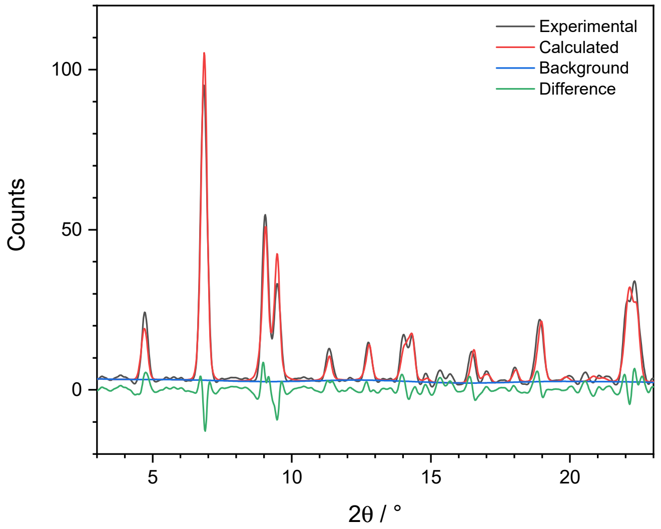

Figure S5. The X-ray powder diffraction pattern of **PROD-1** (black) and a simulated powder pattern (red). Unit cell: *a* = 8.98864 Å; *b* = 13.09307 Å; *c* = 19.04513 Å; α = 100.751°; β = 94.884°; γ = 92.052° Cell volume: 2191.00 $Å^3$; Preferred orientation on plane: 0 1 0; G-factor: 0.8982776; Rp = 17.827; Rwp = 23.606. The cell was indexed using DICVOL06 and fitting was accomplished using Le Bail methods as implanted in EXPO2014.[1–3] (1) Boultif, A.; Louër, D. *Powder Pattern Indexing with the Dichotomy Method. J. Appl. Crystallogr.* **2004**, *37*, 724-731. https://doi.org/10.1107/S0021889804014876. (2) Le Bail, *A. Whole Powder Pattern Decomposition Methods and Applications: A Retrospection. Powder Diffr.* **2005**, *20*, 316–326. https://doi.org/10.1154/1.2135315. (3) Altomare, A.; Cuocci, C.; Giacovazzo, C.; Moliterni, A.; Rizzi, R.; Corriero, N.; Falcicchio, A. *EXPO2013 : A Kit of Tools for Phasing Crystal Structures from Powder Data. J. Appl. Crystallogr.* **2013**, *46*, 1231–1235. https://doi.org/10.1107/S0021889813013113.

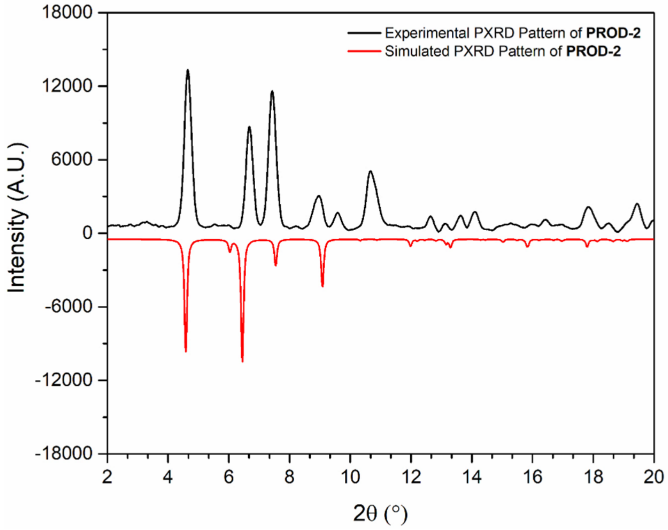

Figure S6. The X-ray powder diffraction pattern of **PROD-2** (black) and a calculated powder pattern based on the single crystal data (red).

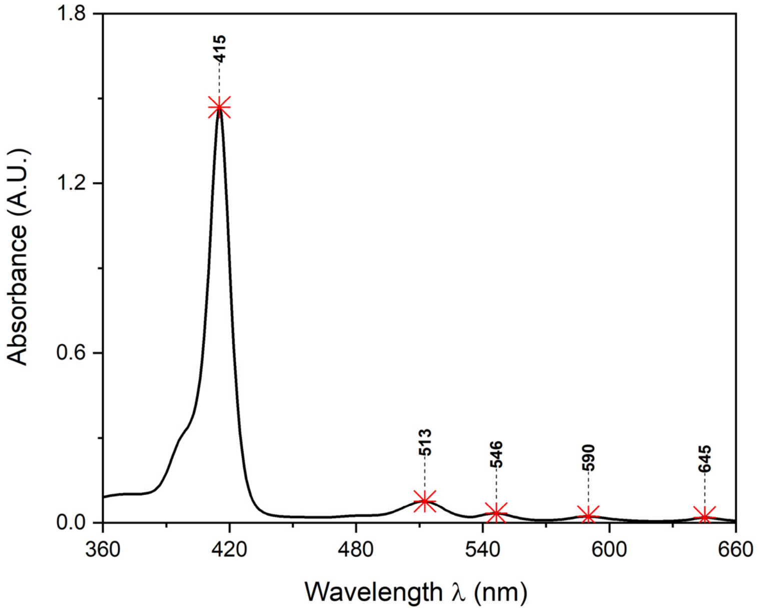

Figure S7. UV-Vis spectrum of 5,15-bis(4-carbomethoxyphenyl)-10,20-diphenylporphyrin recorded in DMF.

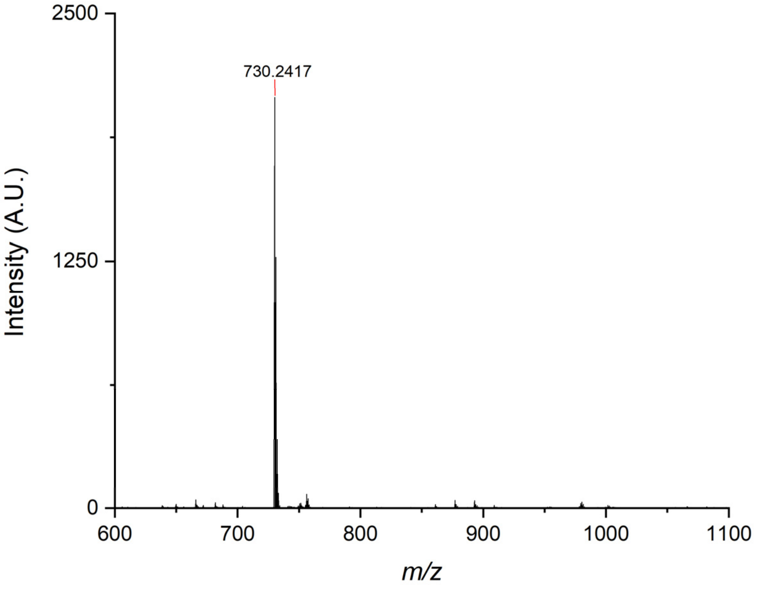

Figure S8. MALDI-TOF MS of 5,15-bis(4-carbomethoxyphenyl)-10,20-diphenylporphyrin. Calculated *m/z* for [C48H34N4O4] = 730.2580.

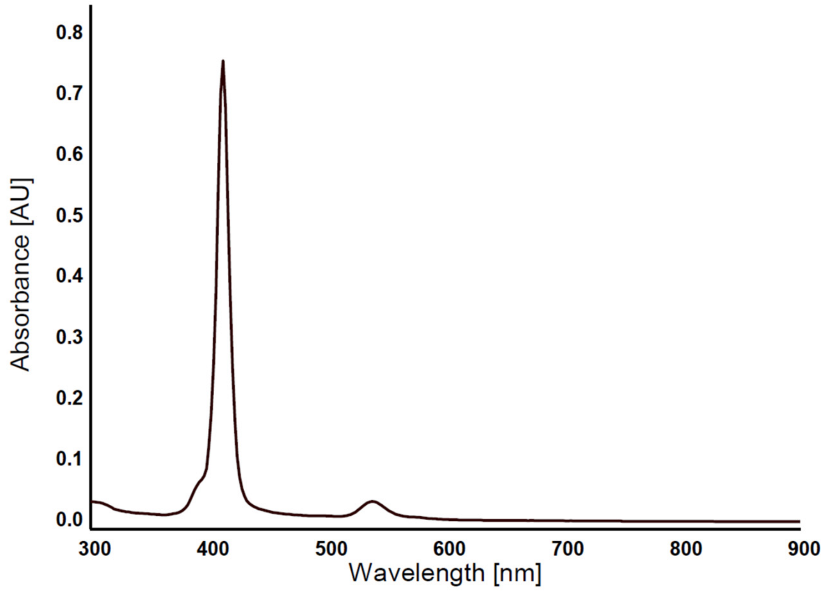

Figure S9. UV-Vis spectrum of [5,15-bis(4-carbomethoxyphenyl)-10,20-diphenylporphyrinato]-Cu^{II} (H_2**L**-Cu^{II}) in MeOH.

Single Mass Analysis
Tolerance = 10.0 PPM / DBE: min = -1.5, max = 200.0
Element prediction: Off
Number of isotope peaks used for i-FIT = 3

Monoisotopic Mass, Odd and Even Electron Ions
29 formula(e) evaluated with 1 results within limits (up to 50 closest results for each mass)
Elements Used:
C: 0-46 H: 0-28 N: 0-4 O: 0-4 Cu: 0-1

Aoife Ryan (MSe), AR969
Q-TOF20150812MF009 25 (0.717) AM (Cen,4, 80.00, Ht,10000.0,1570.68,0.70); Sm (SG, 1x5.00); Sb (15,10.00); Cm (9:30-25)
TOF MS LD+
3.47e+002

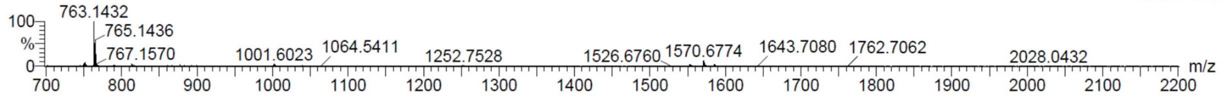

Minimum:				-1.5			
Maximum:		5.0	10.0	200.0			
Mass	Calc. Mass	mDa	PPM	DBE	i-FIT	i-FIT (Norm)	Formula
763.1432	763.1407	2.5	3.3	35.0	23.1	0.0	C46 H28 N4 O4 Cu

Figure S10. MALDI-TOF MS of [5,15-bis(4-carboxyphenyl)-10,20-diphenylporphyrinato]-Cu^{II} (H_2**L**-Cu^{II}).

4. Single-crystal X-ray diffraction

Table S1 – Crystal data and structural refinement parameters for PROD-1.

**PROD-1**	
**Empirical formula**	$C_{52.26}H_{44.03}CuMnN_{4.64}O_{7.07}$
**Formula weight**	968.63
**Temperature/K**	100(2)
**Crystal system**	Triclinic
**Space group**	$P\bar{1}$
**a/Å**	8.98(4)
**b/Å**	14.51(7)
**c/Å**	19.84(10)
**α/°**	108.88(3)
**β/°**	97.60(3)
**γ/°**	95.62(3)
**Volume/Å^3**	2397(19)
**Z**	2
**ρ_{calc}g/cm^3**	1.342
**μ/mm^{-1}**	3.149
**F(000)**	1001
**Crystal size/mm^3**	0.280 × 0.040 × 0.030
**Radiation**	CuKα (λ = 1.54178)
**2Θ range for data collection/°**	4.78 to 137.34
**Index ranges**	-10 ≤ h ≤ 10, -17 ≤ k ≤ 16, -23 ≤ l ≤ 23
**Reflections collected**	8701
**Independent reflections**	8701 [R_{int} = 0.0864]
**Data/restraints/parameters**	8701/15/641
**Goodness-of-fit on F^2**	1.094
**Final R indexes [I>=2σ (I)]**	R_1 = 0.1026, wR_2 = 0.2902
**Final R indexes [all data]**	R_1 = 0.1290, wR_2 = 0.3110
**Largest diff. peak/hole / e Å^{-3}**	1.811 /-0.863

Table S2 – Crystal data and structural refinement parameters for PROD-2.

PROD-2	
**Empirical formula**	$C_{52}H_{39}CoCuN_5O_5$
**Formula weight**	936.35
**Temperature/K**	215(2)
**Crystal system**	monoclinic
**Space group**	*P*2/*c*
**a/Å**	8.668(3)
**b/Å**	14.858(5)
**c/Å**	39.439(14)
**α/°**	90
**β/°**	94.76(2)
**γ/°**	90
**Volume/Å^3**	5062(3)
**Z**	4
**ρ_{calc}g/cm^3**	1.229
**μ/mm^{-1}**	3.473
**F(000)**	1928.0
**Crystal size/mm^3**	0.18 × 0.10 × 0.02
**Radiation**	CuKα (λ = 1.54184)
**2Θ range for data collection/°**	4.496 to 105.11
**Index ranges**	-8 ≤ h ≤ 7, -15 ≤ k ≤ 15, -28 ≤ l ≤ 40
**Reflections collected**	28227
**Independent reflections**	5758 [R_{int} = 0.0486]
**Data/restraints/parameters**	5758/12/578
**Goodness-of-fit on F^2**	1.032
**Final R indexes [I>=2σ (I)]**	R_1 = 0.0632, wR_2 = 0.1766
**Final R indexes [all data]**	R_1 = 0.0793, wR_2 = 0.1893
**Largest diff. peak/hole / e Å^{-3}**	0.471/-1.313

Comments in relation to the crystal structure refinements:

Crystallographic information files for **PROD-1** and **PROD-2** can be obtained free of charge from the Cambridge Crystallographic Data Centre *via* www.ccdc.cam.ac.uk/data_request/cif using the accession identifiers CCDC-**2073036** and CCDC-**2065629**, respectively.

The structures were solved and refined using direct methods with the SHELXTL software package (G. M. Sheldrick, *Acta Cryst. C*, **2015**, *71* ,3-8). For the structural refinement of **PROD-1**, the DFIX constraint was applied to the bonds of an identified coordinated N,N-diethylacetamide (DEA) molecule due its disorder in the structure. Common C-C, C-N and C-O bond lengths, characteristic for DEA molecules were applied. Additionally a FLAT command, restraints some of the atoms of the DEA molecule to lie in a common plane. The approach resulted in convergence upon least-square refinements. Further DEA and MeOH solvent molecules were located in the voids of the structure and their occupancies were refined to achieve convergence. The occupancies <1 are caused by solvent loss during mounting and data collection or disorder of the solvent molecules whereby parts of the disordered positions could not be located. The twin character of the crystals of **PROD-2** was noted during the crystallographic data collection. A careful selection of reflection spots was required prior to indexing and integration. The HKLF5 command was applied to the refinement. This approach allowed us to solve the structure. Finally the Platon twin routine was applied to further resolve the degree of twinning, leading to the reported quality values. The Platon-Squeeze routine was applied due to the diffuse electron density that results from highly disordered solvent molecules located in the voids of the structure. The solvent-accessible void volume accounts to 1143 Å^3 and 272 electrons. This electron contribution stems from solvent molecules, *i.e.* methanol which was used in the synthesis. The TGA analysis is consistent with the crystallographic data. Based on this analysis, 8 constitutional MeOH molecules were assigned to the structure.

Table S3 – Selected Bond lengths [Å] for PROD-1.

Cu(1)-N(2)	2.001(11)
Cu(1)-N(3)	2.003(10)
Cu(1)-N(1)	2.008(9)
Cu(1)-N(4)	2.009(10)
Mn(1)-O(2)#1	2.150(8)
Mn(1)-O(3)	2.155(8)
Mn(1)-O(6)#2	2.157(8)
Mn(1)-O(1)#3	2.172(9)
Mn(1)-O(4)	2.215(10)
Mn(1)-O(5)	2.229(9)

Table S4 – Selected Bond lengths [Å] for PROD-2.

Cu(1)-N(1)	1.978(4)
Cu(1)-N(2)	1.980(4)
Cu(1)-N(4)	1.983(4)
Cu(1)-N(3)	1.983(4)
Co(1)-O(5)	2.265(7)
Co(1)-O(5)#1	2.265(7)
Co(1)-O(1)	2.280(6)
Co(1)-O(1)#1	2.280(6)
Co(1)-O(4)#2	2.372(4)
Co(1)-O(4)#3	2.372(4)
Co(2)-O(3)#4	1.949(4)
Co(2)-O(3)	1.949(4)
Co(2)-O(2)#5	1.958(4)
Co(2)-O(2)#6	1.958(4)
